# Clinical Applicable AI System Based on Deep Learning Algorithm for Differentiation of Pulmonary Infectious Disease

**DOI:** 10.3389/fmed.2021.753055

**Published:** 2021-12-03

**Authors:** Yu-han Zhang, Xiao-fei Hu, Jie-chao Ma, Xian-qi Wang, Hao-ran Luo, Zi-feng Wu, Shu Zhang, De-jun Shi, Yi-zhou Yu, Xiao-ming Qiu, Wen-bing Zeng, Wei Chen, Jian Wang

**Affiliations:** ^1^Department of Radiology, The First Affiliated Hospital of the Army Medical University (Southwest Hospital), Chongqing, China; ^2^Deepwise Artificial Intelligence (AI) Lab, Deepwise Inc., Beijing, China; ^3^Department of Radiology, Huangshi Central Hospital, Affiliated Hospital of Hubei Polytechnic University, Edong Healthcare Group, Huangshi, China; ^4^Department of Radiology, Chongqing University Three Gorges Hospital, Chongqing, China; ^5^Department of Radiology, Chongqing Three Gorges Central Hospital, Chongqing, China

**Keywords:** pulmonary infectious disease, COVID-19, deep learning, computed tomography, pneumonia

## Abstract

**Objective:** To assess the performance of a novel deep learning (DL)-based artificial intelligence (AI) system in classifying computed tomography (CT) scans of pneumonia patients into different groups, as well as to present an effective clinically relevant machine learning (ML) system based on medical image identification and clinical feature interpretation to assist radiologists in triage and diagnosis.

**Methods:** The 3,463 CT images of pneumonia used in this multi-center retrospective study were divided into four categories: bacterial pneumonia (*n* = 507), fungal pneumonia (*n* = 126), common viral pneumonia (*n* = 777), and COVID-19 (*n* = 2,053). We used DL methods based on images to distinguish pulmonary infections. A machine learning (ML) model for risk interpretation was developed using key imaging (learned from the DL methods) and clinical features. The algorithms were evaluated using the areas under the receiver operating characteristic curves (AUCs).

**Results:** The median AUC of DL models for differentiating pulmonary infection was 99.5% (COVID-19), 98.6% (viral pneumonia), 98.4% (bacterial pneumonia), 99.1% (fungal pneumonia), respectively. By combining chest CT results and clinical symptoms, the ML model performed well, with an AUC of 99.7% for SARS-CoV-2, 99.4% for common virus, 98.9% for bacteria, and 99.6% for fungus. Regarding clinical features interpreting, the model revealed distinctive CT characteristics associated with specific pneumonia: in COVID-19, ground-glass opacity (GGO) [92.5%; odds ratio (OR), 1.76; 95% confidence interval (CI): 1.71–1.86]; larger lesions in the right upper lung (75.0%; OR, 1.12; 95% CI: 1.03–1.25) with viral pneumonia; older age (57.0 years ± 14.2, OR, 1.84; 95% CI: 1.73–1.99) with bacterial pneumonia; and consolidation (95.8%, OR, 1.29; 95% CI: 1.05–1.40) with fungal pneumonia.

**Conclusion:** For classifying common types of pneumonia and assessing the influential factors for triage, our AI system has shown promising results. Our ultimate goal is to assist clinicians in making quick and accurate diagnoses, resulting in the potential for early therapeutic intervention.

## Introduction

Pneumonia is a leading cause of death, with mortality among older individuals (70 years) increasing by 33.6 percent between 2007 and 2017 (1). Bacterial pneumonia, viral pneumonia, fungal pneumonia, and parasitic pneumonia are the four types of pneumonia (2), each of which requires different treatment and has a varied prognosis. Rapid pathogen detection and identification are critical for guiding prompt and successful pneumonia therapies, resulting in faster clinical benefits, fewer problems, and lower hospital costs. The existing pneumonia pathogen testing method has various flaws, including low sensitivity and accuracy, long wait times, and high labor expenses. Non-specific medications, such as broad-spectrum antibiotics, might worsen sickness, and raise hospital expenses (3). More effective diagnostic methods with improved accuracy are required to reduce over-treatment.

Computed tomography (CT) plays an important role in the diagnosis of pneumonia. In the lack of a specific image clinical presentation, identifying pneumonia pathogens early and precisely is a major issue (4). Because the imaging signs of different types of pneumonia are similar, making it difficult for radiologists to identify and distinguish them with the naked eye. Furthermore, radiologists' inter-rater variability may result in conflicting outcomes. Artificial intelligence (AI) technologies, particularly deep learning (DL), offer a promising solution for such medical image interpretation, rapid identification, and classification, which can not only avoid doctor heterogeneity but also rapidly and automatically achieve higher diagnostic accuracy. Recent work using AI for the automated diagnosis of pneumonia has also yielded promising results (5–8). In pediatric chest X-rays, DL was used to identify and discriminate between bacterial and viral pneumonia (9, 10). Other studies (5, 11) used CT images to build DL models to identify COVID-19 and distinguish it from community-acquired pneumonia (CAP) and other lung diseases. However, because these studies were designed to focus solely on COVID-19 and normal CT, additional pneumonia manifestations such as bacterial pneumonia were not examined. The real-world situation, on the other hand, would not be similar to this setting. Furthermore, these studies only looked at the image manifestations of pneumonia and ignored the accompanying clinical factors. CT, in conjunction with clinical presentation, can produce a high detection result. Moreover, these approaches do not provide an interpretative study of the model's learning factors, and the prediction models that arise may not be useful in guiding early and quick identification of various pulmonary infections. Some studies (5, 9–11) utilized class activation maps (12), a sort of heat map that overlays CT scans to indicate the important areas for model predictions. Although intuitive, these heat maps do not offer radiologists useful information for describing features or interpreting for fundamental clinical indications.

CT characteristics, also referred to as key imaging features or clinical indicators, include the number, location, and extents of different pulmonary lesions, such as ground-glass opacity (GGO) and consolidation. In recent studies of COVID-19 pneumonia, some of these CT characteristics, like lesions, have been exploited to monitor the progress of diseases (13). In contrast, others, like lesion location, were found to be risk factors for poor outcome (14). Although such accurate and automated quantification of these CT characteristics has already been made possible by machine learning-based algorithms, few studies have made efforts to assist radiologists in understanding the predicted results produced by the systems.

In this retrospective study, we aimed to develop and validate a CT-based DL system to classify pneumonia patients into four pathogenic types: common virus, bacteria, fungus, and SARS-CoV-2. This method will facilitate faster diagnosis and subsequently, more suitable treatment for pneumonia patients. Furthermore, we retrieved a slew of quantitative CT features or clinical indications, such as lesion numbers and location. In order to help radiologists in interpreting CT scans of pneumonia patients, we evaluated the relative relevance of each imaging feature in determining the pathogenic sources of pneumonia in a standard machine learning (ML) model/classifier.

## Methods

### Patient Cohort and Data Collection

The ethics committees approved this multi-center retrospective study and written informed consent was waived because the data used for system development were de-identified by removing personal information. Patients with respiratory symptoms suggestive of pulmonary infection (fever, cough, and sputum production) were enrolled in this research, who underwent chest CT scanning and received laboratory confirmation of the underlying pathology of pneumonia: SARS-CoV-2, common virus, bacterium, or fungus. The four pathogens of pneumonia were identified using reverse transcriptase-polymerase chain reaction (RT-PCR) and culture and microscopic inspection of sputum, blood, or lung tissue samples. From January 2011 to February 2020, we gathered 7,487 anonymous lung CT images from 2,195 individuals using these first criteria. Then, individuals who had previously undergone thoracic surgery, had severe TB, or had no radiological indications of pneumonia were eliminated. We also eliminated individuals with respiratory artifacts, less than three slices, or a thickness more than 3 mm on their CT images.

**Figure 1:**
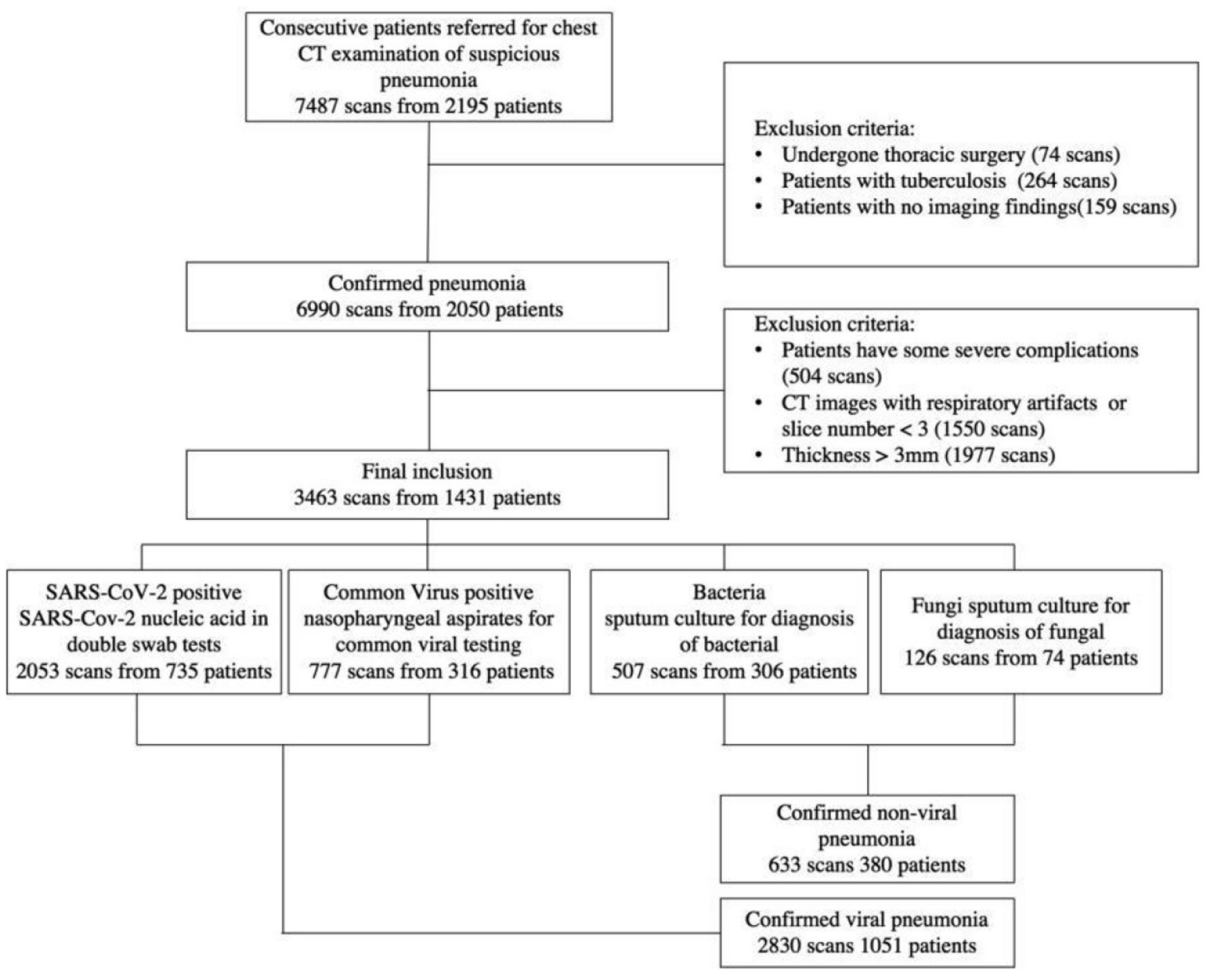
A flow diagram of the patient selection process with inclusion and exclusion criteria is shown. Between January 2011 and February 2020, this study included 2,195 patients from three institutions, 1,431 of whom were finally used to construct the classification system.

Finally, a total of 1,431 patients from three institutions were employed in this study to establish the classification system. Figure 1 has more information on the inclusion and exclusion criteria, as well as a flowchart. To evaluate the robustness of our AI system in various clinical settings, the CT data obtained in this study came from a range of vendors, including Toshiba Medical Systems, Japan; GE Healthcare, USA; United Imaging, China; and Siemens Healthineers, Germany. All CT scans were obtained with a resolution of 512^*^512 with slice spacing ranging from 0.625 to 3 mm in the axial direction. A tube voltage of 120 kVp was used for CT examinations. The automated tube current modulation approach was utilized to control the tube current (30–70 mAs). The examinations were carried out in helical mode with a helical pitch of 0.8125–0.984 mm.

### Overview of the AI System

**Figure 2:**
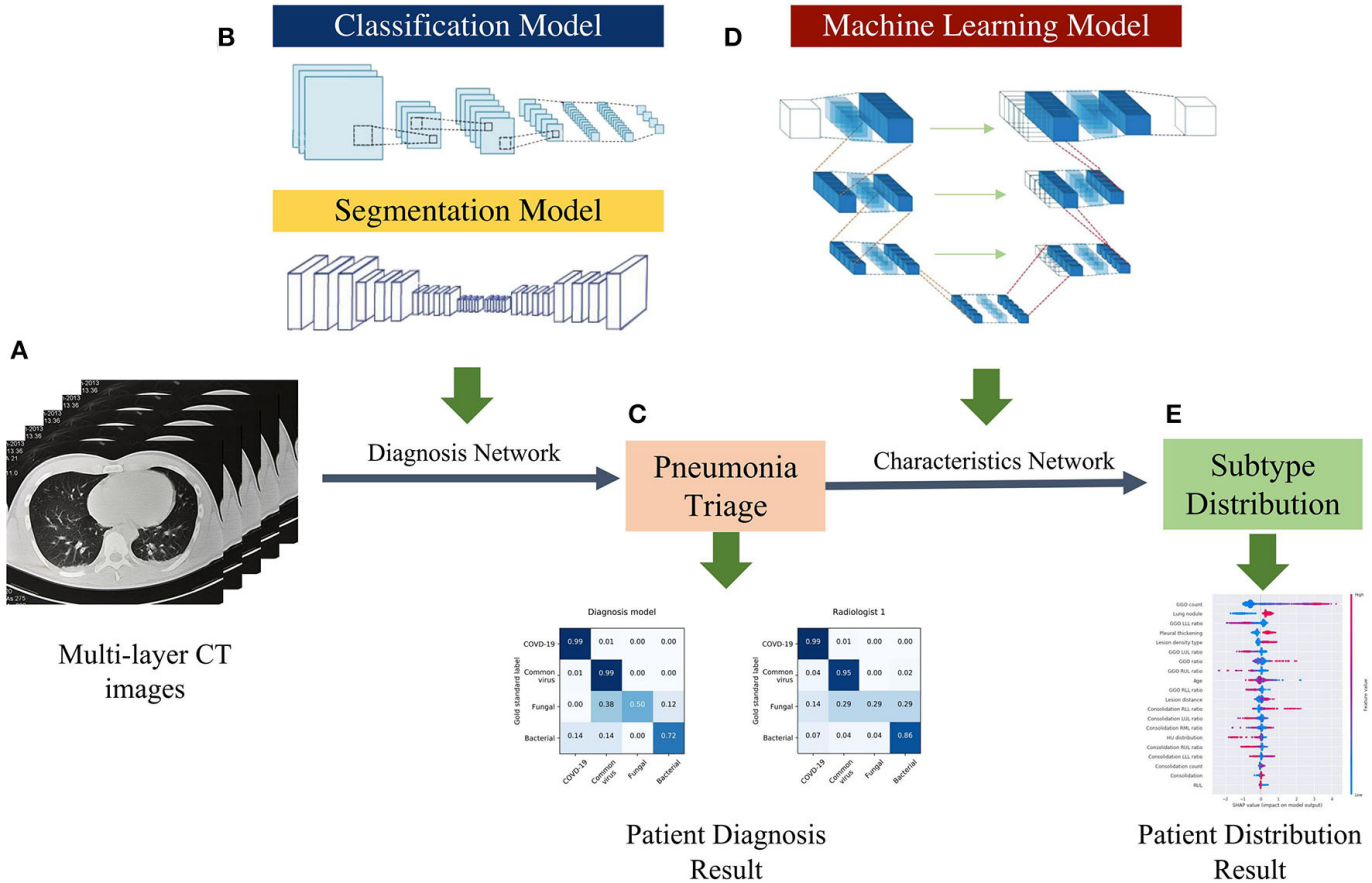
AI framework of the study. **(A)** Using multi-layer raw pneumonia CT images and associated etiology labels as input, the data will be flipped horizontally as data augmentation to increase the number of training samples and reduce the possibility of over-fitting. **(B)** The proposed DL system is composed of two models: a classification model and a segmentation model. The classifier can detect abnormal slices and predict pathogen type scores, while the segmentation model extracts CT image features (lung lobe and the contour of the lesions). **(C)** The voting schema calculates the ratio of particular positive slices and votes on their patient-wise triage base on the image-wise diagnosis score. **(D)** The ML system is trained with the clinical factors and CT features quantified by the DL system. **(E)** Patients CT factors and clinical features distribution and probabilities evaluated by characteristics network to assist radiologists in understanding the predicted results produced by the systems.

We proposed a four pathogenic classification AI system for pneumonia that uses CT images as input and explains the interactions between the factors learned by the model (image and clinical records) to help clinicians make accurate and efficient predictions (Figure 2). The suggested classifier consists of three tasks, the first two of which were trained using a deep learning model (DL system) with a convolution neural network based on the PyTorch frame [(15); Figure 2B], and the third by a machine learning method (ML system) (Figure 2D). Based on radiologists' recommendations, the first two CNN classifiers were developed: a bi-classifier for differentiating viral from non-viral pneumonia and a quad-classifier for the four pathogenic types. The given data was split into three sets with an 8:1:1 ratio for training, validation, and testing, while the third task combining images and medical records information to explain the clinical indicators.

#### Construction of the Deep Learning System

The pipeline of our deep learning-based system included four key components: (a) an abnormal-slice identification model (normal or abnormal), (b) a segmentation model that segmented the lung lobe and the contour of the lesions, (c) a classification model that investigated multiple indicators of pneumonia and differentiated the types (bi-classifier or quad-classifier), and (d) a voting model that merged the CT slices-wise scores to generate a patient-level CT volume prediction. The abnormal CT slices with pneumonia-related lesions were used to train a convolutional neural network (CNN)-based classifier for the pneumonia pathogens. Specifically, for CT volumes, we have developed modified ResNet-50 networks (16) for radiological abnormality identification. We also developed a novel lesion segment network architecture for contour extraction of lesions and lobes, based on the trained backbone parameters and further fusing the extracted feature (lesion size, counts) to imitate physician diagnostic practice. In order to extract 3D context information based on a given lesion slice, this module used continuous multi-slice CT images as input to learning the weights of different layers and adaptive modifies network learning parameters depending on spatial changes in lesions. Furthermore, the model was designed for multi-resolutions, and the information gathered at various resolutions is adaptable in order to provide a more complete information basis on lesions of varying sizes. The high cost of data collection and labeling influences the difficulty of modeling pneumonia framework. As a result, transfer learning was used to solve the problem of insufficient training data by first learning the specific weights of the neural network on the source data set such as ImageNet (17) and then re-learning the appropriate weights for some of the different instances of the target data set. By majority voting, the final score of the CNN classifier's prediction for all abnormal CT slices was merged to generate a patient-level CT volume prediction. In the validation cohort, we preprocessed the given CT scan in the same way that we did in the training cohort. After that, the preprocessed image is sent to the backbone for predictions and majority voting. The code for reproducing the study's findings is available at https://github.com/chiehchiu/CAAS.

#### Construction of the Machine Learning System

The DL system was built just to evaluate medical images, neglecting the complementary nature of medical records and visuals, as well as the need to see and comprehend the issue from several viewpoints. A written medical record reflects on the patient's health, and the image of the patient depicts the condition using the pathogenesis idea. The combination of both improves the patient's overall condition and reduces misdiagnosis.

To provide a comprehensive diagnosis of the image's clinical and case information, our machine learning-based system analyzes all data samples obtained from image and quantitative CT characteristics such as GGO count and location, as well as other clinical indicators such as sex and age, and to explain the interactions between the factors learned by the model. We utilized Shapley Additive exPlanation (SHAP) (18) on the XGBoost classifier (19) to analyze the contribution of each feature in detecting pneumonia pathogens (Figure 2D). The most important step in this model is the filtering of key features. We need to filter several of the features obtained in the previous step (quantitative CT characteristics) to remove those that may cause model deviation and those with low correlation. The specific methods are as follows: (a) screening based on statistical features such as variance; (b) using the maximum correlation and minimum redundancy feature selection methods and the lasso feature selection method to regress the highly correlated features of the predicted target and obtain the key features with high stability, discrimination, and independence; and (c) based on the lasso feature selection method to get the best K features for preservation. To counteract the class imbalance in our dataset during model training, we also used down-sampling and over-sampling as needed.

### Expert Performance Assessment

Two groups of doctors with varying levels of experience (three junior radiologists [3–4 years of experience] and three senior radiologists [7–8 years of experience]) were asked to evaluate pneumonia cases solely on CT scans independently and blindly to establish a comparative baseline for our AI system. In group examinations of three physicians, annotated lesions were identified as positive samples whereas the lesions viewed by two or more radiologists were considered as true lesions.

### Statistical Analysis

The following measures were used to assess the performance of our classifiers: area under the receiver operating characteristic curve (AUC), accuracy, sensitivity, and specificity (20). The DeLong technique (21) was used to calculate the 95% confidence intervals (CIs) for the AUC. The median and interquartile range (IQR) with a 95% confidence interval (CI) are used to represent continuous variables. The ANOVA test was used to determine whether there was a difference between the two or four pathogenic categories of pneumonia patients (22). For categorical characteristics, the χ^2^ or Fisher exact test (23) was employed to compare the pathogenic groups. All statistical tests were two-tailed, with statistical significance set at *p* < 0.05.

## Results

### Study Population Characteristics

The study cohort included 1,431 laboratory-confirmed pneumonia patients with 3,463 chest CT scans, with 316 patients with 777 scans having viral pneumonia, 306 patients with 507 scans having bacterial pneumonia, 74 patients with 126 scans having fungus pneumonia, and 735 patients with 2,053 scans having COVID-19. The study comprised 779 men (49.6 ± 15.6; 14–94 years) and 652 women (48.9 ± 14.8; range, 15–90 years). According to the data split strategy, 2,990 CT series with 492,346 slices were utilized for training, 255 CT series with 41,825 slices were used for validation, and 218 CT series were used for testing (Table 1).

**Table 1:** Summary of training + validation and testing datasets by four pathogenic types.

	**Training** **+** **validation**					**Testing**				
	**COV-19**	**Common**	**Bacterial**	**Fungal**	***P*-value**	**COV-19**	**Common**	**Bacterial**	**Fungal**	***P*-value**
Patients	688 (51.4%)	293 (21.9%)	287 (21.4%)	70 (5.2%)	–	47 (50.5%)	23 (24.7%)	19 (20.4%)	4 (4.3%)	–
Scans	1,925 (59.3%)	722 (22.2%)	480 (14.8%)	118 (3.6%)	–	128 (58.9%)	55 (25.1%)	27 (12.3%)	8 (3.7%)	–
Slices	374,862	89,310	55,328	14,671	–	40,446	7,630	3,159	1,216	–
Male	386 (56.1%)	154 (52.5%)	152 (52.9%)	37 (52.8%)	0.75	24 (51.1%)	13 (56.5%)	11 (57.9%)	2 (50.0%)	0.17
Age ≥60 years old	144 (20.9%)	129 (44.0%)	122 (42.5%)	27 (38.6%)	–	9 (19.1%)	9 (39.1%)	8 (42.1%)	2 (50.0%)	–
Age <60 years old	544 (79.1%)	173 (56.0%)	165 (57.5%)	43 (61.4%)	–	38 (80.9%)	14 (60.9%)	11 (57.9%)	2 (50.0%)	–
Mean age	47.6 ± 14.7	55.2 ± 16.2	56.6 ± 14.3	52.8 ± 18.8	<0.001	45.8 ± 14.6	57.9± 15.0	58.9 ± 13.8	52.1± 21.4	<0.001

*Data presented as n (%) unless otherwise indicated. Mean ages are reported as mean ± standard deviation. COV-19, COVID-19 pneumonia; common, common virus pneumonia*.

### Deep Learning-Based Pathogen Identification

The performance of our pneumonia pathogens classification system was assessed on test data and described in Table 2. The first level of the diagnostic system categorized the virus pneumonia (SARS-CoV-2, common virus) and non-virus pneumonia (bacterium, fungus). The proposed bi-classifier achieved an average AUROC of 0.984 (95% CI, 0.983–0.985) on slice-level and 0.988 (95% CI, 0.977–0.997) on patient-level, respectively. The performance results (cut-point yield maximum specificity plus specificity) showed a sensitivity of 0.931 (95% CI, 0.926–0.937), specific of 0.945 (95% CI, 0.943–0.947), accuracy of 0.939(95% CI, 0.937–0.941) for slice-level and sensitivity of 0.959 (95% CI, 0.899–0.988), specific of 0.965 (95% CI, 0.938–0.992), accuracy of 0.961 (95% CI, 0.937–0.941) for patient-level. Then, within each categorized system, further sub-classifications and hierarchical layers were made, where applicable. Our quad-classifier also performed better on classifying SARS-CoV-2 and common virus [AUROC: 0.995 (0.990–0.998) and 0.986 (0.977–0.995)] than bacteria and fungus [AUROC: 0.984 (0.970–0.995) and 0.991 (0.978–1.000)], especially in terms of sensitivity of 0.978 (95% CI, 0.946–1.000) and specificity of 0.947 (95% CI, 0.915–0.977), which included both common non-viral pneumonia and viral pneumonia cases as binary distracters.

**Table 2:** The performance of the DL system in making multi-pathogenic types classification based on the CT cohort.

	**Metric**	**Bi-classifier**			**Quad-classifier**				
		**Mean**	**Viral**	**Non-viral**	**Mean**	**COV-19**	**Common**	**Bacterial**	**Fungal**
Testing	AUC (95%CI)	0.984 (0.983, 0.985)	0.980 (0.979, 0.981)	0.988 (0.987, 0.989)	0.985 (0.983, 0.987) (slice-level)	0.983 (0.982, 0.984)	0.987 (0.986, 0.988)	0.990 (0.989, 0.991)	0.979 (0.974, 0.984)
	Accuracy (%)	0.939 (0.937, 0.941)	0.926 (0.924, 0.928)	0.953 (0.951, 0.954)	0.937 (0.934, 0.938)	0.931 (0.929, 0.933)	0.932 (0.930, 0.934)	0.938 (0.936, 0.940)	0.944 (0.942, 0.946)
	Sensitivity (%)	0.931 (0.926, 0.937)	0.916 (0.914, 0.920)	0.945 (0.938, 0.954)	0.961 (0.955, 0.968)	0.918 (0.915, 0.922)	0.989 (0.985, 0.993)	0.981 (0.975, 0.987)	0.957 (0.943, 0.971)
	Specificity (%)	0.945 (0.943, 0.947)	0.937 (0.934, 0.940)	0.953 (0.951, 0.955)	0.938 (0.936, 0.941)	0.946 (0.943, 0.949)	0.927 (0.925, 0.929)	0.936 (0.934, 0.938)	0.943 (0.941, 0.946)
Testing	AUC (95%CI)	0.988 (0.977, 0.997)	0.993 (0.986, 0.998)	0.983 (0.968, 0.996)	0.989 (0.978, 0.997) (patient-level)	0.995 (0.990, 0.998)	0.986 (0.977, 0.995)	0.984 (0.970, 0.995)	0.991 (0.978, 1.000)
	Accuracy (%)	0.961 (0.936, 0.979)	0.959 (0.932, 0.977)	0.963 (0.941, 0.982)	0.954 (0.933, 0.977)	0.968 (0.954, 0.991)	0.959 (0.941, 0.982)	0.932 (0.900, 0.959)	0.959 (0.936, 0.977)
	Sensitivity (%)	0.959 (0.899, 0.988)	0.952 (0.924, 0.977)	0.964 (0.875, 0.999)	0.978 (0.946, 1.000)	0.984 (0.969, 1.000)	0.965 (0.919, 1.000)	0.964 (0.897, 1.000)	1.000 (1.000, 1.000)
	Specificity (%)	0.965 (0.938, 0.992)	0.967 (0.937, 0.999)	0.963 (0.937, 0.984)	0.947 (0.915, 0.977)	0.946 (0.900, 0.988)	0.957 (0.933, 0.981)	0.927 (0.892, 0.962)	0.958 (0.933,0.976)

**Figure 3:**
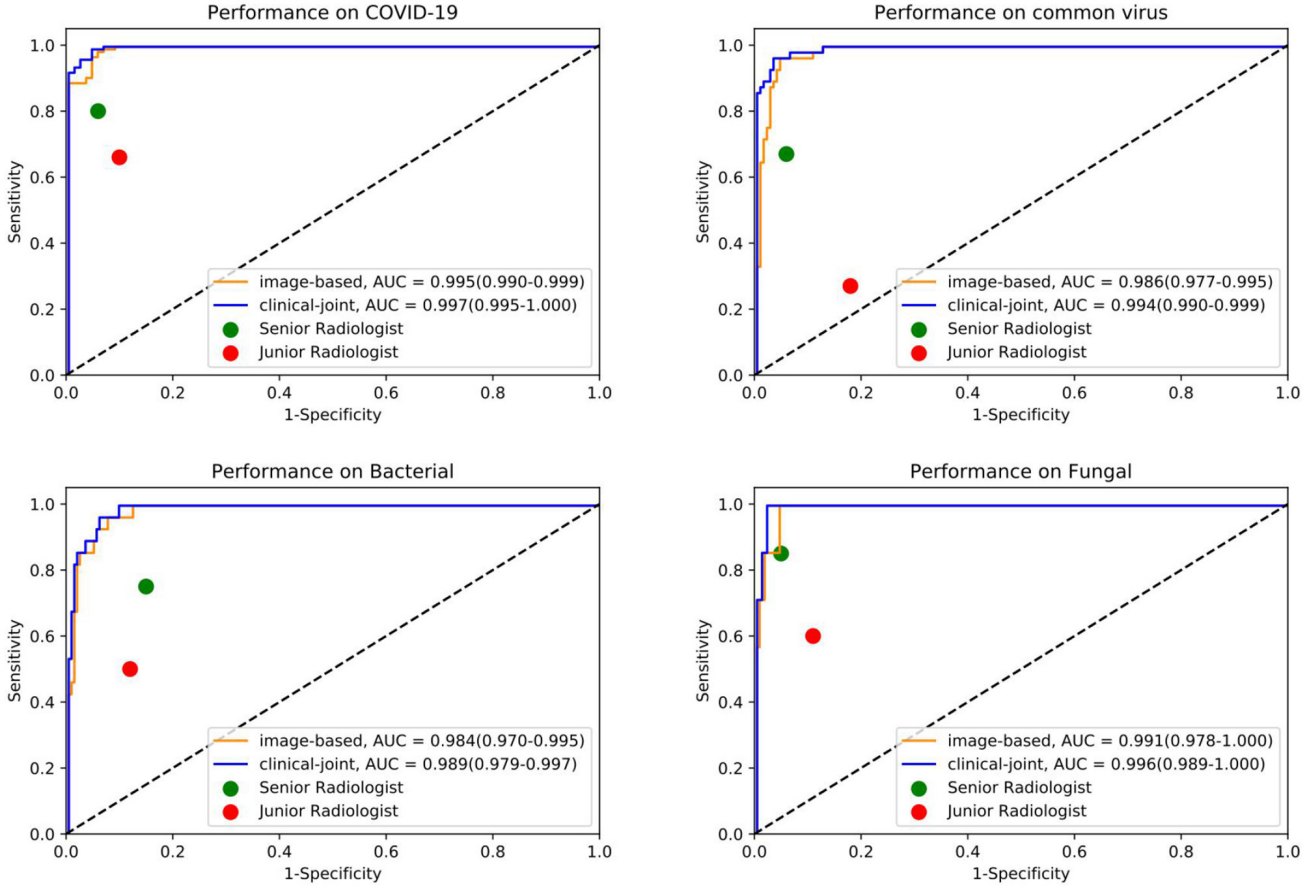
The individual ROC curves of our image-based DL system and clinical-joint ML system in classifying the four pathogens of pneumonia on testing dataset. In the observer performance test, the AI system performed much better than all reader groups in terms of four type classification.

To identify positive cases, we also established cutoff values of the output probability value based on the findings, resulting in a high-sensitivity cutoff of 98% sensitivity for patient-wise classification and a high-specificity cutoff of 98% specificity. From this result, operating thresholds were defined as a probability of 0.15 [high sensitivity threshold; sensitivity, 0.979 (95% CI, 0.977–0.981); specificity, 0.795 (95% CI, 0.790–0.801)] and 0.91 [high specificity threshold; sensitivity, 0.836 (95% CI, 0.828–0.846); specificity, 0.980 (95% CI, 0.980–0.982)]. We plotted the AUC curves of our quad-classifier on each pathogenic category, as shown in Figure 3, which also showed a similar trend.

### Machine Learning-Based Feature Analysis

In this study, we utilized machine learning (ML) algorithms to integrate chest CT results (quantified by a DL system) with clinical symptoms in order to promptly diagnose patients who tested positive for four forms of pneumonia (Table 3). Then, using these features, determining the contribution of outcome to the prediction of pneumonia types. On the test set, we assessed the ML models and compared their performance to that of a DL system and two groups of radiologists with varying levels of expertise. The AUROC were calculated for both human readers and the two models in Figure 3 and Table 4. The ML algorithm showed a satisfactory performance with an AUC of 0.997 (95% CI, 0.995–1.000) for SARS-CoV-2, 0.994 (0.990–0.999) for common virus, 0.989 (0.979–0.997) for bacteria, and 0.996 (0.989–1.000) for fungus. The senior radiologist using both the CT and corresponding clinical data achieved a 80.6% sensitivity (95% CI 76.4%, 84.7%; *P* = 0.0510), 93.8% specificity (95% CI 88.5%, 97.1%; *P* = 0.005) for Covid-19. The junior radiologist fellow using both the CT and clinical data achieved a 66.0% sensitivity (95% CI 57.1%, 74.5%; *P* < 1 × 10–4), 90.3% specificity (95% CI 84.3%, 94.6%, *P* = 0.090). *P*-values indicate the significance of difference in performance metric compared with respect to the joint model.

**Table 3:** Lesion characteristics in CT image of different types of pneumonia.

	**Metric**	**COV-19**	**Common**	**Bacterial**	**Fungal**	***P*-value**
Characteristics	Patients	670	268	241	70	–
	Age (year)	47.5 + 14.7	55.7 + 15.0	57.0 + 14.2	52.8 + 18.9	0.6
	Sex (male)	371 (55.4%)	142 (53.0%)	126 (52.3%)	37 (52.1%)	0.80
Total lesion percent (%)	–	3.5 (7.9%)	15.7 (24.1%)	5.7 (11.4%)	7.2 (17.0%)	<0.001
GGO percentage in each lung lobe (%)	LUL (%)	3.4 (9.2%)	12.1 (20.5%)	3.3 (9.2%)	4.8 (13.1%)	<0.001
	LLL (%)	2.7 (9.2%)	16.2 (23.8%)	4.6 (12.7%)	4.8 (13.8%)	<0.001
	RUL (%)	1.7 (7.3%)	10.6 (19.8%)	2.4 (6.6%)	3.8 (12.5%)	<0.001
	RML (%)	1.3 (6.7%)	8.4 (17.6%)	0.5 (2.9%)	2.7 (10.6%)	<0.001
	RLL (%)	2.7 (9.4%)	14.0 (21.7%)	2.8 (8.8%)	3.3 (10.8%)	<0.001
Consolidation percentage in each lung lobe (%)	LUL (%)	6.8 (14.5%)	9.4 (21.5%)	4.3 (9.4%)	12.1 (19.2%)	<0.001
	LLL (%)	13.9 (21.1%)	13.8 (26.7%)	16.0 (20.0%)	12.9 (21.5%)	0.33
	RUL (%)	8.7 (16.8%)	11.2 (23.6%)	6.2 (10.0%)	13.9 (21.5%)	<0.001
	RML (%)	8.6 (18.3%)	9.1 (20.6%)	3.1 (10.2%)	11.1 (21.5%)	<0.001
	RLL (%)	16.3 (22.8%)	14.4 (25.6%)	14.9 (18.1%)	17.4 (25.5%)	0.17
Density: HU distribution within lesions	>−200 HU (%)	9 (0.4%)	43 (6.5%)	14 (4.0%)	9 (4.8%)	0.12
	−400~−200 HU (%)	96 (4.8%)	65 (9.8%)	68 (19.3%)	44 (23.4%)	0.25
	−600~−400 HU (%)	678 (34.0%)	264 (40.0%)	171 (48.4%)	84 (44.7%)	<0.001
	< −600 HU (%)	1,209 (60.7%)	287 (43.5%)	100 (28.3%)	51 (27.1%)	<0.001
Location: distance from lesion to pulmonary pleurae	Lesion distance (mm)	4.8 ± 4.3	1.3 ± 2.6	2.2 ± 3.4	3.0 ± 3.3	<0.001

*GGO, ground glass opacity; RUL, right upper lobe; RML, right middle lobe; RLL, right lower lobe; LUL, left upper lobe; LLL, left lower lobe; HU, hounsfield unit. Data presented as n (%) unless otherwise indicated. Lesion distance are reported as mean ± standard deviation*.

**Table 4:** Receiver operating characteristic (ROC) of the Image-based model and Clinical-joint model.

	**Image-based model**	**Clinical-joint model**	***P*-value**
COV-19	0.995 (0.990, 0.998)	0.997 (0.995, 1.000)	0.032
Common	0.986 (0.977, 0.995)	0.994 (0.990, 0.995)	0.018
Bacterial	0.984 (0.970, 0.995)	0.989 (0.979, 0.997)	<0.001
Fungal	0.991 (0.978, 1.000)	0.996 (0.989, 1.000)	<0.001

**Figure 4:**
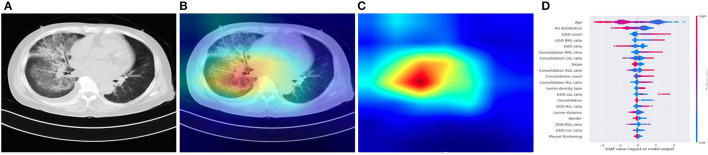
Illustration of characteristics that contribute to the prediction of pneumonia using CAM and SHAP. **(A)** A COVID-19 patient's origin image sample. **(B,C)** Visualize the attention regions of a network for distinct abnormality and disease categories. **(D)** The relative contribution of each CT or clinical measure to predicting the probability of pneumonia prediction. Features to the right of the risk explanation bar increased the danger, while features on the left decreased it.

Regarding clinical features interpreting (Table 3), there are no significant difference in terms of sex (*p* = 0.80) and age (*P* = 0.6). The four pathogenic groups differed in most of the CT characteristics (*p* < 0.001; Figure 4). Patient's age, lesion features such as GGO count, presence of lung nodule, and lesion density type, were significant features associated with SARS-CoV-2 status. The GGO features were identified as the most significant contributor to the evaluation of identifying COVID-19 from the four pneumonia types [odds ratio (OR), 1.76; 95% CI: 1.71–1.86; *P* = 0.003]. Clinical parameters relating to the lesion location (right upper lung or, 1.12; 95% CI: 1.03–1.25, *P* = 0.01) contributed to the prediction of viral pneumonia patients.

## Discussion

In this study, we presented an effective clinically relevant AI system based on medical image identification and clinical feature interpretation system based on real-world datasets. The accuracy of our AI system for distinguishing the four common types of pneumonia were relatively high [COVID-19 (99.7%), common viral pneumonia (99.4%), bacterial pneumonia (98.9%), and fungal pneumonia (99.6%)]. Furthermore, using a specialized CT analysis, we retrieved dozens of quantitative CT features from the study cohort as CT findings or clinical indications. The GGO characteristics were found as the most important contributors in identifying the four pneumonia types. Notably, the COVID-19 patients had more GGO lesions; patients with common viral pneumonia were less likely to have bilateral lung infection; and patients with fungal pneumonia had a modest number of consolidation lesions. In this study, we present an AI system that outperforms immediate-level radiologists on differentiating the pulmonary infection based on CT scans. This fast imaging-based triaging system has the potential to be a non-culture technique for identifying common pneumonia, which would promote timely targeted antibiotic treatment for pneumonia patients and thus help reduce antimicrobial resistance, treatment side effects, and costs. During the COVID-19 pandemic, this system can also help stratify pneumonia patients for proper care or quarantine and thus lessen the burden of diagnosing numerous potentially infected patients. With the availability of more fine-grained pneumonia data, this system can easily be extended to recognize new strains or sub-strains of pulmonary infections.

Our DL system performed well in differentiating the four major kinds of pneumonia, and our results are somewhat more accurate than the prior AI study-based CT for COVID-19 diagnosis (24). Although CT is an essential tool for early detection of pneumonia, it is not as accurate in identifying the virus in the absence of clinical symptoms. In ML system, our joint AI model incorporates CT and clinical data, demonstrating that clinical information played a role in the accurate diagnosis of pneumonia in individuals in the early stage. Compared with radiologists, our CT image-based AI system can identify the possible pathogenic infectious pneumonia more quickly, and the accuracy is much improved, to timely guide clinical medication to maximize the patients' benefits. The timeliness and accuracy of AI can not only enable patients to get correct treatment decisions at an early stage, reduce hospitalization duration, and save treatment costs, but also significantly reduce the incidence of complications caused by delayed diagnosis and treatment decisions because of waiting for pathogen detection (25). The quantitative CT characteristics extracted from the study cohort by a dedicated CT analysis can help physicians to interpret better the CT scan and the prediction made by our system, such as more GGO lesions in the COVID-19, less bilateral lung infection in common viral pneumonia, fungal pneumonia had a moderate amount of consolidation lesions. The SHAP explainer also supported this statistical observation on the XGBoost classifier built from these CT features, which revealed the top 20 most important CT characteristics for predicting the pathogens of pneumonia, including age, GGO ratio, lesion position, and consolidation. The listing of these CT features together with their relative importance to the pathogen classification provides a clinician instant valuable information, instead of a straight diagnosis suggestion, of a chest CT scan that can help them make an informed decision on the final diagnosis and treatment. It can also serve as a training tool for junior radiologists to interpret CT scans and make a better judgement.

The GGO features were identified as the most significant contributor in identifying the four pneumonia types. GGO has traditionally been non-specific and can be seen in all types of pneumonia (26, 27), but a recent study has found subtle differences in GGO between different diseases (28). Our study found that there were statistically significant differences in the distribution of GGO among the four pneumonia types, showing that our AI system could distinguish subtle differences in GGO from the four pneumonia types.

Our AI system combines the clinical advantages of CT and the intelligent advantages of AI, and has a good application prospect in clinical practice. In contrast to etiological tests such as RT-PCR, CT has some advantages. Although RT-PCR is the gold standard, but it also has certain instability. RT-PCR and other etiological tests can only make exclusive and definite diagnosis in the diagnosis of pneumonia, that is, RT-PCR can only detect covid-19 infection; CT, on the other hand, can simultaneously identify a variety of pathogens in the diagnosis process. And the detection rate of RT-PCR and other etiological tests is susceptible to some factors, such as variation in detection rate from different manufacturers, low patient viral load, or vulnerable clinical sampling (29), and so on. RT-PCR is prone to false negative results and may require repeated testing (30),So, compared with RT-PCR, CT is more economical and faster, and the CT scan showed more stable results and higher sensitivity (31).

Although our AI system was more accurate than human experts in this aspect, and have many advantages, it cannot completely replace the gold standard set by laboratory tests. Future research could be conducted to address the issues as mentioned earlier. For instance, our AI system can benefit from more data samples in the bacteria and fungus groups. Clinical or laboratory information (such as exposure history and blood biochemical examination) may be incorporated as an additional information source into our CT-based AI system to boost the classification accuracy.

There are some limitations in our research. Firstly, the incidence of fungal pneumonia is much lower than that of other pneumonia, so the data volume of fungal pneumonia is much smaller than that of other pneumonia, in subsequent studies, we will further expand the data of fungal pneumonia. Secondly, our data contains different examinations of the same patient at one admission, but there is no different scan reconstruction of the same examination. The reasons for this are as follows: (1) The amount of data is small; (2) For a certain patient, examinations that took place at different time were included in our study. For different examinations of the same admission, CT findings will present different characteristics according to different phases of the course of the disease. Therefore, training and testing with CT images of the same pathogen infection at different periods (progression/improvement) can improve the performance and robustness of the model. This approach might have some limitations regarding to metric calculation, but other studies (5) have also adopted a similar approach. Moreover, compared to their method, our data have broader inclusion criteria and are more in line with real clinical scenarios. When we built the model, the data of training set and test set were randomly selected, which would not have a great influence on the final result. And thirdly, due to geographical and other factors, we cannot obtain data from other countries, so we only conduct data analysis on Chinese patients. This does have some limitations. But we are willing to disclose the code release: (https://github.com/chiehchiu/CAAS), and very welcome more countries researchers use more diversified data for research.

In conclusion, we proposed a CT-based AI system that can assist clinicians in classifying patients into four pathogenic types efficiently and accurately by listing quantitative CT characteristics and their importance for making the prediction. This study takes the first step in developing a rapid, CT-based, non-culture diagnostic method to triage pneumonia patients for timely targeted treatment. The proposed classifier may be used in pre-screening patients to conduct triage and fast-track decision making before RT-PCR.

## Data Availability Statement

The data used in this work is subject to the following licenses/restrictions: data sets cannot be made public. Access to these datasets should be requested through Wei Chen at cwjxl_2006@163.com or Jian Wang at wangjian_811@yahoo.com.

## Ethics Statement

The studies involving human participants were reviewed and approved by the Ethics Committees of the First Affiliated Hospital of Army Medical University, PLA (Approval Number: KY2020036). Written informed consent for participation was not required for this study in accordance with the national legislation and the institutional requirements.

## Author Contributions

JW, WC, Y-hZ, and X-fH all helped to conceptualize and design the study. X-mQ and W-bZ recruited patients. X-qW and H-rL sorted out the data. This manuscript was primarily written by Y-hZ, X-fH, and J-cM. Y-hZ, X-fH, J-cM, Y-zY, Z-fW, SZ, D-jS, WC, and JW analyzed the data. Y-hZ, X-fH, J-cM, SZ, WC, and JW provided feedback on prior drafts of the work. The final manuscript was read and approved by all writers.

## Funding

This work was supported in part by the Central Government Guided, the local special fund for Science and Technology Development, the local Science and Technology Innovation Projects (WX2017-07-05), the National Innovation Talent Promotion Program (4139Z2399), and the Beijing Municipal Science and Technology Planning Project (Grant No. Z211100003521009).

## Conflict of Interest

J-cM, Z-fW, SZ, D-jS, and Y-zY were employed by company Deepwise Inc. The remaining authors declare that the research was conducted in the absence of any commercial or financial relationships that could be construed as a potential conflict of interest.

## Publisher's Note

All claims expressed in this article are solely those of the authors and do not necessarily represent those of their affiliated organizations, or those of the publisher, the editors and the reviewers. Any product that may be evaluated in this article, or claim that may be made by its manufacturer, is not guaranteed or endorsed by the publisher.
